# Work Shift and Circadian Rhythm as Risk Factors for Poor Sleep Quality in Public Workers from Murcia (Spain)

**DOI:** 10.3390/ijerph17165881

**Published:** 2020-08-13

**Authors:** María Teresa Rodríguez-González-Moro, José Miguel Rodríguez-González-Moro, José Miguel Rivera-Caravaca, Tomás Vera-Catalán, Agustín Javier Simonelli-Muñoz, Juana Inés Gallego-Gómez

**Affiliations:** 1 Faculty of Nursing, Catholic University of Murcia (UCAM), 30107 Murcia, Spain; mtrodriguez@ucam.edu (M.T.R.-G.-M.); tvera@ucam.edu (T.V.-C.); jigallego@ucam.edu (J.I.G.-G.); 2 Department of Pneumology, Hospital Universitario Príncipe de Asturias, Alcalá de Henares, 28805 Madrid, Spain; respirama@yahoo.es; 3 Department of Cardiology, Hospital Clínico Universitario Virgen de la Arrixaca, Instituto Murciano de Investigación Biosanitaria (IMIB-Arrixaca), CIBERCV, 30120 Murcia, Spain; jmrivera429@gmail.com; 4 Liverpool Centre for Cardiovascular Science, University of Liverpool, Liverpool L7 8TX, UK

**Keywords:** sleep quality, circadian rhythm, public workers, work shift

## Abstract

The purpose of this study was to determine the prevalence of sleep quality and to investigate variables predicting the risk of poor sleep quality in public workers from Murcia (Spain). A cross-sectional and prospective study was conducted from October 2013 to February 2016 in 476 public workers. The Pittsburgh Sleep Quality Index was used to measure the quality of sleep, and the reduced scale of the Horne and Österberg Morningness–Eveningness Questionnaire was applied to analyze the circadian typology. The predictive variables of self-reported poor sleep quality were identified by multivariate logistic regression. No significant differences were found according to sex in the overall sleep quality scores (5 ± 2.9 versus 5.1 ± 3, *p* = 0.650), but there were in the duration of sleep. Three percent of females slept <5 hours compared to 2% of men (*p* = 0.034). Fixed morning shifts (OR = 1.9, 95% CI 1.3–3.1; *p* = 0.007) and evening chronotypes (OR = 1.6, 95% CI 1.0–2.3; *p* = 0.017) were independent predictors of suffering from poor sleep quality. In conclusion, the frequency of self-reported poor sleep quality among public workers from Murcia was 37.4%. Being a public worker with a fixed morning shift and having an evening chronotype demonstrated to be associated with the quality of sleep.

## 1. Introduction

Sleep is central in individuals’ health and the quality of sleep is of great importance. Lack of sleep directly affects cognitive abilities and states of mood. This situation causes exhaustion, and it is related to stress resistance, work capacity, reaction time, and concentration [1]. Thus, a good quality of sleep in middle-aged workers is associated with a better performance of tasks, a greater ability to be focused on work as well as lower interpersonal conflicts and errors in execution. On the contrary, lack of sleep, going to sleep late, and poor sleep quality are predictors of fatigue and stress at work [2] as well as burnout [3] and greater interference in daily chores in a worker’s home [4].

The natural rhythms of day and night influence all living beings. The body of the human being is synchronized with a cycle called circadian rhythm. Hence, the level of activation, metabolism, temperature, heart rate, and hormonal activity increase and decrease depending on this internal clock. These activities reach their peak during the day and decrease dramatically at night while sleeping. A third of this rhythm happens during sleep with the sleep–wake cycle being the main of all circadian rhythms [5]. This cycle is generated in the suprachiasmatic nucleus and changes its activity essentially by modifications of light, resulting in the basis of the “biological clock” of the individual [6].

Previous studies on sleep deprivation have verified that the appearance of sleep depends not only on the time that the person has been awake, but also on the circadian rhythm that induces periodic changes in the level of vigilance. Those who have not slept the night before do not feel progressively sleepier during the day, but changes in the level of vigilance are produced as a direct consequence of circadian oscillations [7].

Nowadays, there have been gradual changes in the sleep patterns of individuals. The increase in exposure to artificial light at night, nightlife, work shifts, travel, and long-haul flights as well as the excessive use of electronic screens are just some examples of factors involved in these changes. As a consequence, the prevalence of sleep disorders has increased thus making its study more important. At work, optimal performance is demanded, assuming that individuals achieve a good sleep quality, and that during working hours, they are able to work with maximum efficiency. However, the rising of nighttime recreational activities and the increasing demand by the society of rotating work shifts to maintain their activity for 24 hours lead workers to sleep during the day in addition to shorter periods of sleep during the night, affecting the quality of sleep overall and preventing them from performing their work correctly [8]. Many of the workers fulfilling these conditions are public workers. Nevertheless, most studies on the relationship between sleep quality and work shifts have focused primarily on healthcare providers and variable work shifts [9,10]. For this reason, it is necessary to analyze this relationship in public, non-sanitary workers with different shifts including those with fixed mornings, since the evidence on how work shifts affect the quality of sleep of such workers is scarce [11].

The aim of this study was to determine the prevalence of self-reported sleep quality and to investigate those factors that may predict the risk of suffering poor sleep quality in a large sample of public workers from Murcia.

## 2. Materials and Methods 

### 2.1. Design and Sample

A descriptive–observational, cross-sectional, and prospective study on public workers of the Autonomous Community of the Region of Murcia (Spain), dependent on the Occupational Risk Prevention Service of the General Directorate of Public Function and Quality of Services, was carried out. Workers from technical positions with university qualification, manual laborers (security guards, assistants, cooks, electricians, etc.), administrative positions, and management positions were considered suitable for the study. They were recruited based on a consecutive non-probabilistic sampling procedure. 

The software Ene 2.0 (GlaxoSmithKline, Brentford, UK) was used to calculate the sample size based on an estimation of 55.7% of poor sleep quality obtained from other studies [12] with an accuracy of ±5%, an α error of 5%, and for an infinite population. A minimum sample of 380 public employees was necessary. 

The self-administered questionnaire was carried out by public workers who received periodic health screenings from October 2016 to February 2019. 

The study was approved by the Ethics Committee from the Catholic University of Murcia (UCAM, code: CE061409), and all subjects signed informed consent (10.2% refused to participate). Several meetings were held to explain to the medical staff the characteristics and purpose of the research, guaranteeing the anonymity of the participants according to the Declaration of Helsinki [13].

### 2.2. Measures

Participants were given a questionnaire that included the Pittsburgh Sleep Quality Index (PSQI) and the reduced scale of the Horne and Österberg Morningness–Eveningness Questionnaire. The PSQI evaluates participants’ sleep quality and determines potential sleep disorders. It is composed of 19 items (arranged into seven components) that analyze different factors determining the quality of sleep (C1–C7): subjective quality of sleep (C1); latency of sleep (C2); duration of sleep (C3); usual sleep efficiency (C4); frequency of disorder (C5); use of hypnotic medication (C6); and daytime dysfunction (C7). Each component ranges from 0 (no difficulty) to 3 points (serious difficulty). The sum of these components represents the overall sleep quality; the higher score, the worse quality. A global result ≥5 categorizes subjects as bad sleepers [14]. In the Spanish population, the sensitivity and specificity are 88.63% and 74.14%, respectively [15].

For the analysis of the circadian typology or chronotype, we used the reduced scale of the Horne and Österberg Morningness–Eveningness Questionnaire by Adan and Almirall [16] which consists of 5 questions on fundamental aspects of circadian rhythmic expression, for example, the preferred time to get up or go to bed and the level of activation upon waking. A higher score is indicative of a morning chronotype [16].

In addition, personal descriptive variables such as sex and age were analyzed. Work shift was categorized as “variable shift” (2 mornings (7:30 a.m.–3:30 p.m.), 2 afternoons (3:00 p.m.–10:00 p.m.), and 1 night (10:00 p.m.–8:00 a.m.); resting then for 3 days) or “fixed morning shift” (7:30 a.m.–3:30 p.m.).

### 2.3. Analytic Strategy

The different variables were expressed as the mean ± standard deviation for continuous variables and absolutes frequency with percentage (%) for categorical variables. For the bivariate analysis, the Student’s t-test, one-way ANOVA, Pearson’s chi-square, and Pearson’s correlation coefficient were used, when appropriate. To assess variables independently associated with the risk of self-reported poor sleep quality, a multivariate logistic regression analysis was performed, using the stepwise selection method. The odds ratio (OR) with its corresponding 95% confidence interval (95% CI) was obtained for each variable. 

A *p*-value < 0.05 was selected as statistically significant. The data were analyzed using the SPSS statistical software for Windows (SPSS Inc., Chicago, IL, USA).

## 3. Results

We included 476 public workers. The mean age was 47.5 ± 7.2 years with ages between 25 and 66 years. The majority of subjects (51.3%) were males, mostly technicians with a university qualification (59%) and with a fixed morning shift (76.5%).

The mean score in the reduced scale of Morningness–Eveningness Questionnaire by Adan and Almirall [16] was 17.8 ± 3.06 points. According to the scale, 285/460 (62.0%) public workers had a morning chronotype and 175/460 (38.0%) were classified as having an evening-intermediate chronotype (Table 1).

The mean PSQI score obtained was 5.03 ± 2.9 points, and 37.4% (178) of public workers were qualified as poor sleepers (Table 1). The proportion of patients in each of the seven components of the PSQI is shown in Table 2. In C1 “subjective quality of sleep”, 20% of the subjects described it as “bad” or “very bad”. As for “sleep latency” (C2), 35.3% of the workers needed “less than 30 minutes” to fall asleep, 49.6% needed “between 31 and 60 minutes”, and 15.1% needed “more than one hour”.

The mean score in the reduced scale of the Morningness–Eveningness Questionnaire was similar between females and males (17.9 ± 3.1 versus 17.7 ± 3.1; *p* = 0.311). Similarly, the PSQI score was not different in females and males (5.1 ± 3.1 versus 5.0 ± 2.9; *p* = 0.650). However, we found statistically significant differences according to sex in sleep duration (C3). Thus, 80.6% of females slept more than 6 hours in contrast to 82.0% of men, and 3.0% of females slept less than 5 hours compared to 2.0% of males (*p* = 0.034). Full results of this analysis are shown in Supplementary Materials Table S1.

Most workers with self-reported poor sleep quality had a fixed morning shift (82.6%), whereas only 17.4% of workers reporting poor sleep quality had a variable shift (82.6 versus 17.4%, *p* = 0.015). Indeed, workers with a morning chronotype reported poorer sleep quality, finding a negative and significant correlation between the reduced scale of Morningness–Eveningness Questionnaire and the PSQI (*r* = −0.1; *p* = 0.042). Therefore, the higher the morning profile, the better the quality of sleep.

To predict the risk of self-reported poor sleep quality, a multivariate logistic regression analysis was performed which showed that workers with fixed morning shift had an almost two-fold higher risk of suffering poor sleep quality than those with variable shifts (OR = 1.90; 95% CI 1.19–3.12; *p* = 0.007). On the other hand, the evening chronotype was also independently associated with poor sleep quality (OR = 1.60; 95% CI 1.08–2.34; *p* = 0.017).

## 4. Discussion

The first relevant result that we found in this study was that more than one-third of public workers from Murcia reported poor sleep quality. A previous study showed a similar proportion of poor sleepers in Spain (44.3%) [12], and a systematic review and meta-analysis concluded that, overall, the prevalence of poor sleep quality was 39% in the adult population [17]. In our opinion, this is of great interest, since poor quality of sleep has negative impacts on individuals’ professional lives causing fatigue, loss of attention, concentration and reducing motivation and efficiency at work [9], in addition to being related to symptoms of depression and anxiety [18], obesity [19], type 2 diabetes, [20,21] and metabolic syndrome [22].

On the other hand, the proportion of poor sleep quality found in our study could be in relation to the fact that the total sleep hours was less than 5 hours in 18.7% of workers, mainly in females compared to males. Indeed, it has been shown that subjects sleeping less than 6 hours have lower cognitive performance [23]. This is essential in a work environment where working time, competitiveness, and the continuous search for more trained personnel are increasing, while habits and the duration of sleep time are progressively getting worse. A night of lack of sleep significantly affects the function of the hippocampus which imposes a deficit in the ability to retain new learning in memory [24].

On contrary what was described in previous studies, we found that the majority of public workers with poor sleep quality were on morning work shifts, and morning fixed work shifts were predictors of poor sleep quality. When investigating the possible reasons, we found studies that related sleep quality to sleep latency [25]; however, only 15.1% of our public workers recognized problems falling asleep in the last month with a latency exceeding 30 minutes. Another potential origin was introduced by Rasslazova et al. [26]. According to these authors, a high intention to sleep leads to fragmentation of sleep, increasing in nighttime awakenings, and the rate of excitement, also leading to marginally significant reductions in total sleep time. Workers with fixed morning shifts should get up early, and they use to go to bed with too much awareness and intention to sleep to rest the necessary hours thus producing the opposite effect. Hence, starting work early, for example, at seven o’clock in the morning, has a negative influence on sleep hygiene. In a large sample of Swedish workers with a fixed morning shift, Akerstedt et al. [11] found that with the increasing advance of the work start time, the time of waking up also advanced, but bedtime changed very slightly. Thus, early work start times were not compensated with earlier bedtimes and total sleep time decreased as the work start time got earlier. Similarly, Van de Ven et al. [27] found an association between the start time of the work shift and the sleep complaints with a greater need for recovery when the shift began before 7:00 a.m. in the morning. Souza et al. [28] demonstrated that teachers with morning shifts starting classes at 7:00 a.m. in the morning suffered worse sleep quality than those who had classes in the afternoon. 

Another reason could be the controversial “siesta” (i.e., nap) so extended in Mediterranean countries. Workers on fixed morning shifts tend to have naps in the afternoon after lunch. A nap of more than half an hour can cause alterations in the natural biological rhythm, promoting subsequent insomnia [29]. In addition, although naps seem to compensate for the poor subjective quality of sleep and, to some extent, the short duration of sleep, it must be considered that afternoon naps reduce the duration of nighttime sleep on the same day [30]. According to the foundation of health education of the San Carlos Clinical Hospital in Madrid [31], the average duration of a nap in Spain is 1 h, which is higher than recommended.

Along the same lines, it is important to note that the broadcast of the programs with the largest television audience (prime time) is later in Spain than in the rest of Europe, delaying dinner time and bedtime. Therefore, the commission for the rationalization of Spanish schedules advocates a change in habits to improve productivity, including the variation of television schedules, since there has been a progressive delay in the broadcast of these programs, conditioning a delay in the start of sleep [32].

All of the above factors could cause alterations in the circadian rhythm and the chronotype. Both influence different areas of people’s functioning, highlighting implications for performance and mental health. The morning people (those who always have work in the morning) usually go to bed and get up earlier, and their optimal performance occurs during the morning they are usually more synchronized with the solar cycle [33,34], while the evening people (those who tend to work in the afternoon) go to bed and get up later, optimizing their performance in the afternoon or evening [34]. The morning typology is more frequent until the age of 10 and from the age of fifty, and increases with age [34,35]. Although in the present study we found an important interaction between the evening chronotype and sleep quality, Yıldırım and Boysan [36] using signal detection analysis could not find a significant association between circadian preferences and sleep quality in healthy individuals from Turkey. Similarly, Antúnez, Navarro and Adan in a systematic review [37] reported that there were no differences in time or quality of sleep between evening and morning individuals, although the latter tended to get up and go to bed two or three hours earlier according to polysomnographic records if they were free to choose schedules. However, if subjects with an evening chronotype had to get up early, it was less frequent that they felt asleep; sleep had a shorter duration and a nap was more usually required. 

As a limitation of the study, it should be noted that only subjective tests were used to measure sleep quality and it would also be convenient using objective tests such as actigraphy. In addition, it would be interesting to expand the sample to other Autonomous Communities of Spain in future studies. 

## 5. Conclusions

The frequency of self-reported poor sleep quality among public workers in Murcia was 37.4%. Public workers with fixed morning shifts and evening chronotypes reported poorer sleep quality and these factors were actually independent risk factors of substantially increasing poor sleep quality. Administrations should encourage a change of habits and create greater awareness about the effects of poor sleep quality on fixed shift public workers in Murcia.

## Figures and Tables

**Table 1:** Baseline characteristics of the public workers included.

Variables	*N* or Mean	% or SD
**Age (years), Mean (SD)**	47.5	7.2
**Sex**		
Male	244	51.3
Female	232	48.7
**Work Position**		
Managers	59	12.4
Technicians with university qualification	281	59.0
Administrative	99	20.8
Manual laborer	37	7.8
**Work Shift**		
Variable shift	112	23.5
Fixed morning shift	364	76.5
**Self-Reported Sleep Quality**		
Good quality of sleep	298	62.6
Poor quality of sleep	178	37.4
**Circadian Rhythm**		
Morning profile	285/460 *	62.0
Evening-intermediate profile	175/460 *	38.0

* 16 workers did not answer the Morningness–Eveningness Questionnaire.

**Table 2:** Distribution of public workers in each of the seven components of the Pittsburgh Sleep Quality Index.

PSQI Score	C1	C2	C3	C4	C5	C6	C7
	Subjective Quality of Sleep (%)	Latency of Sleep (%)	Duration of Sleep (%)	Usual Sleep Efficiency (%)	Disturbances (%)	Use of Hypnotic Medication (%)	Daytime Dysfunction (%)
0 = very good	29.8	35.3	37.8	75.4	5.3	85.7	45
1 = good	50.2	49.6	43.5	17.3	73.7	6.9	45
2 = bad	17.6	12.2	16.2	5.5	20	2.1	10.1
3 = very bad	2.3	2.9	2.5	1.9	1.1	5.1	2.9

## Supplementary Materials

The following are available online at https://www.mdpi.com/1660-4601/17/16/5881/s1. Table S1: Comparison of each of the seven components of the Pittsburgh Sleep Quality Index between male and female public workers.
