# Supplementary material for: Work Shift and Circadian Rhythm as Risk Factors for Poor Sleep Quality in Public Workers from Murcia (Spain)
Source: Int J Environ Res Public Health. 2020 Aug 13;17(16):5881. doi: 10.3390/ijerph17165881 (PMC7459974; doi:10.3390/ijerph17165881)
Supplement: Supplementary file 1 [file ijerph-17-05881-s001.pdf]

Supplementary Material

Supplementary Table 1. Comparison of each of the seven components of the Pittsburgh Sleep Quality Index between males and females public workers.

PSQI score		C1 Subjective quality of sleep	p-value	C2 Latency of sleep	p-value	C3 Duration of sleep	p-value	C4 Usual sleep efficiency	p-value	C5 Disturbances	p-value	C6 Use of hypnotic medication	p-value	C7 Daytime dysfunction	p-value
0 = very good	Male (%)	27.9	0.538	37.3	0.414	32.4	0.034	73.4	0.277	6.1	0.650	87.3	0.508	50.4	0.038
	Female (%)	31.9		33.2		43.5		77.5		4.3		84.4		39.2	
1 = good	Male (%)	52.5		50.0		49.6		17.2		74.6		5.3		37.7	
	Female (%)	47.8		49.1		37.1		17.3		72.8		8.7		46.6	
2 = bad	Male (%)	18.0		9.8		16.0		6.6		18.4		2.5		10.2	
	Female (%)	17.2		14.7		16.4		4.3		21.6		1.7		9.9	
3 = very bad	Male (%)	1.6		2.9		2.0		2.9		0.8		4.9		1.6	
	Female (%)	3.0		3.0		3.0		0.9		1.3		5.2		4.3	
